# Acute Laminitis1Read before the meeting of the Wisconsin Society of Veterinary Graduates, Madison, February 5, 1897.

**Published:** 1897-06

**Authors:** B. L. Clark

**Affiliations:** Monticello, Wis.


### ACUTE LAMINITIS.[1]

By B. L. Clark, M.D.C.,
MONTICELLO, WIS.

The subject I have chosen is acute laminitis or inflammation of the sensitive laminæ of the foot. I do not expect to present anything new, as this disease has been thoroughly investigated and dis-

[1] Read before the meeting of the Wisconsin Society of Veterinary Graduates, Madison, February 5, 1897.

cussed in works on veterinary pathology, but simply to refresh our minds on the subject. This disease is quite common among horses, and is frequently seen in cattle. It is the most-dreaded disease to which the horse is liable. It is not confined to the feet alone, although its chief seat is there. It has a variety of causes—as over-exertion, error in feeding, drinking cold water when heated, standing in a constrained position for a long time, a sudden chill, or indigestion. The last cause in my opinion is the most common. It frequently arises from other diseases, or, rather, is a complication of such diseases as pneumonia, enteritis, or bronchitis, where the whole surface of the body is affected, as seen in cases where the hair of the mane and tail falls out. The tendency of this disease is to throw off the hoof, as indicated by the rings on the external surface of the hoof-wall. It occasionally arises from over-exertion and concussion, exemplified in cases where horses are run against time, but it is much more frequent where the mucous membrane is affected. In cases of concussion the pedal bone is involved, and results in ostitis, and sometimes necrosis and sloughing of the hoof, terminating in an agonizing death. The symptoms of both forms are almost identical.

*Pathology.* Inflammation of the sensitive foot includes the laminæ, sole, and os pedes, resulting in an exudation which is greatest at the toe, the tissues being more vascular at that part. The pain is most agonizing and persistent, owing to the fact that the foot is enclosed in an unyielding horny box. The bloodvessels being engorged and having no chance to swell or expand, pressure upon the anterior part of the pedal bone has a tendency to force it downward and separate it from the wall. As the result of the inflammation there is an exudation thrown out at this point which is of a dirty reddish color; this fills the canals and lacunæ of the pedal-bone, forming a barrier to the circulation of the blood, thus hastening the process of necrosis and decay. Should the inflammation persist the exudation accumulates at the toe, separating the horny and the sensitive laminæ. As a result of this the anterior part of the os pedis is forced downward, producing a convex condition of the sole, while the secreting power of the sole is arrested or prevented, and the horny sole becomes weak and spongy, affording but little protection to the sensitive parts within. The outer horn of the wall or that secreted by the coronary band now presents a ribbed appearance, as if attempts had been made to cast off the hoof. These rings are irregular and run together at the anterior part of the hoof. Prof. Williams says that occasionally we have suppuration at the coronet and sometimes at the sole, detaching the sensitive from the horny sole. Laminitis produced by concussion is usually confined to the front feet.

Many writers state that affections of serous membranes, such as the pleura, frequently terminate by metastasis in laminitis. But I am of the opinion that when the sensitive laminæ become involved during the progress of some other disease it is always the mucous membrane that is affected, owing to the fact above-mentioned, that the skin is continuous with the mucous membrane; it is also involved and in this way extends to the feet. The skin being affected is somewhat congested, and will naturally involve all of its appendages. The circulation of the feet being restricted within an unyielding space, the walls being unable to expand, thus produce the intense pains, by the engorged bloodvessels pressing upon the nerves. We also have a fair example of their relation in cutaneous eruptions from indigestion, catarrh, etc.; the irritation may be very slight, scarcely observable. But the irritation of the folds of the sensitive laminæ in the foot surrounded by its unyielding case causes the acute symptoms.

Acute laminitis may terminate in resolntion of the parts, suppuration, gangrene, and subacute or chronic laminitis. The chronic form of the disease is seen when the acute has partly subsided and the symptoms somewhat abated. Horses suffering with chronic laminitis are predisposed to the acute form. The pathological changes of the chronic are the descent of the os pedis, thus causing a convex, weak sole, and a great horny growth at the toe.

*Causes.* Indigestion, concussion, bad shoeing, hereditary tendency, and defective conformation. It is said by some that horses with wide feet are more liable to the disease. But it is the general opinion that horses with moderately small feet, and weighing about twelve to thirteen hundred pounds, are most often affected. The exciting causes are: over-exertion, concussion, and indigestion when caused by an engorged stomach. The disease is generally confined to the front feet, especially when caused by concussion. But when caused by indigestion it may affect the hind feet only, or all four of the feet.

*Symptoms.* The horse is excessively lame and almost immovable, especially at starting; the whole body seems to be cramped. He stands with hind feet well under the body, and front ones advanced to relieve them from the weight as much as possible. If all four feet are affected he will put them nearly together, sway backward and forward, and from side to side in order to get the weight on the heel as much as possible, the toes being elevated from the ground.

If compelled to move he elevates his feet with difficulty; he will often groan with pain and sweat freely in patches, while the remainder of the surface of the skin will be hot and dry. Temperature from 102° to 105° F.; pulse full, hard, and increased in frequency; the general symptoms are those of agonizing pain. Appetite is somewhat impaired and thirst generally increased. They usually maintain the standing position, but when they do lie down they often remain for some time, and seem to be relieved. Cases of suppuration and gangrene are not very frequent owing to the anatomical structure of the veins of the foot. The feet will be found to be hot to the touch, and when tapped with the hammer the animal will show signs of pain. The pulse at the digital artery will be found full and throbbing, the respirations accelerated, the mouth hot and dry, the feces dry and coated, urine scanty and high colored. The symptoms vary with the feet that are affected. When the front feet are affected, which is most frequently the case, they are advanced forward, and the hind feet are carried well under the body to relieve them of their load. But when the hind feet only are involved the animal will often lie down. It may be most marked in one foot, but this is generally due to some local lesion.

In the ox it is more serious in the inner claw on account of it bearing the most weight. He walks with difficulty and takes advantage of every opportunity to lie down. The fever is severe, and sometimes there is loss of appetite and rumination.

*Termination of the acute form.* Well-treated laminitis is of short duration, and terminates in a short time in resolution. If it runs for a longer period than eight to twelve days, chronic laminitis will probably result. Resolution in the acute form is accomplished when the inflammation is quickly subdued and the products are absorbed; the horn-secreting structure of the foot is then not often altered.

*Complications and terminations.* Hemorrhage, inflammation with exudation, suppuration, gangrene, and lastly chronic laminitis.

Hemorrhage is that condition when rupture of the capillaries has taken place, thereby infiltrating between the two laminæ, and sometimes oozing out at the coronet. If oozing has taken place it is advisable to cut in at the toe, at the junction of the wall and sole, thus relieving the pressure.

Inflammation is the result of congestion if not relieved, and as a natural consequence we have exudation, this exudation being thrown out from the sensitive lamina separating the sensitive from the horny, thus pushing the pedal bone downward, and the wall upward, thereby forming a condition known as chronic laminitis.

Suppuration is more rare, and is only seen in the traumatic form. It generally attacks the sensitive sole, and occasionally works its way to the coronet. This is a very painful complication. Relief may be obtained by making an opening at the toe, but sloughing and dropping off of the entire hoof is occasionally seen.

Gangrene of the subhorny tissue sometimes takes place, though seldom, under the influence of excessive pressure, especially when there is a subhorny exudation. When this sets in the violent pains suddenly cease, resting is more solid, and he moves without difficulty; but at the same time the countenance of the patient is anxious and contracted, the pulse becomes small and difficult to count, the temperature diminishes, and the animal has an unsteady gait, is indifferent to excitement, and soon he dies from septic infection.

Chronic laminitis is that condition of the foot when resolution has not taken place in ten to fifteen days.

*Treatment.* Give a mild purgative (aloes I think is best), follow with febrifuges, as aconite, the tr. or fl. ex.; if the fever is high may give every two to four hours. May give potassium nitrate, as it seems to produce good results in this disease. Remove the shoes and stand the horse with affected feet in hot water as deep as the pastern-joint, let him remain there for thirty to forty minutes. Then remove and apply hot, soft linseedmeal poultices. Repeat the bathing two or three times a day, and each time apply a fresh poultice. At the end of about the third day cease the bathing, and change the poultices every twelve hours until relief has been afforded. At about the third or fourth day I think it advisable to give a laxative. Would also advise walking exercise, twenty to thirty minutes three or four times a day. At the onset of the disease the horse should be placed in a loose box-stall. well ventilated, and bedded deeply with short straw or sawdust. If the weather is cold clothe him warmly. If the appetite is not lost give soft, easily digested food. After the effects of the disease have passed off, he may be shod with a wide web shoe, and the external wall and sole of the hoof should be painted three or four times per week with some kind of hoof-ointment. During this time he may be put to moderate work, or better still, if in summer time, turn on wet, low pasture for several months, which will soften and stimulate the growth of the hoof.

---

Users of milk in Terre Haute, Ind., have learned that a so-called preservine, highly injurious when taken into the stomach, is largely used in the milk sold in that city.

